# P-2132. Comparison of Clinical Characteristics and Risk Factors among Adult Patients with Bloodstream Infections due to *candida auris* vs other *candida* Species: a 3-year analysis in a Large Care Tertiary Hospital, Saudi Arabia

**DOI:** 10.1093/ofid/ofae631.2287

**Published:** 2025-01-29

**Authors:** Faisal Alasmari, Asiya Wali, Mahmoud Mukahal, Seham Alsurayhi, Qais Dirar, Hani Tamim

**Affiliations:** King Fahad Medical City, Riyadh, Ar Riyad, Saudi Arabia; King Fahad Medical City, Riyadh, Ar Riyad, Saudi Arabia; King Fahad Medical City, Riyadh, Ar Riyad, Saudi Arabia; King Fahad Medical City, Riyadh, Ar Riyad, Saudi Arabia; Alfaisal University, Riyadh, Ar Riyad, Saudi Arabia; Alfaisal University, Riyadh, Ar Riyad, Saudi Arabia

## Abstract

**Background:**

Candida auris is considered a serious global threat due to its potential to cause invasive infections, staggering transmissibility and mortality. We evaluated the epidemiologic and clinical features of candida bloodstream infections (BSIs) and explored risk factors associated with C. auris.

**Methods:**

A retrospective review of all candida BSI in patients aged ≥ 18 years were conducted during August 2018 to June 2021. C. auris cases were compared to other Candida species cases. Odds ratio (OR), estimated using stepwise regression analysis, was used to assess the association between risk factors and incidence of C. auris infection.

**Results:**

We identified 137 patients with first episode Candida BSIs. 32% patients were secondary to C. auris and the remaining were due to: C.albicans (20%), C.glabrata (18%), C.Parapsilosis (12%), C.trpicalis (15%), and others (4%). 60% of total cohort were male and their age were more than 55 yearsz. The proportions of patients with underlying diseases e.g. diabetes mellitus, hypertension, cardiovascular diseases, chronic renal diseases and COVID-19 infection were not significantly different. The prevalence of malignancy was significantly higher in non C. auris group (P= 0.01). The following risk factors were independently associated with C. auris BSI: Prior Use of antifungals (OR: 3.60, CI: 1.18-10.98), Previous history of multidrug resistant bacterial infection (OR: 3.37, CI: 1.41-8.06), and intensive care unit stay (OR 8.6, CI 2.61-28.37).

**Conclusion:**

The epidemiology of candidemia is changing with non-albicans becoming predominant. Judicious use of all types of antimicrobials including antifungals will help in controlling the emergence of C. auris infection.

**Disclosures:**

All Authors: No reported disclosures

